# Radiolabelling of Polyclonally Expanded Human Regulatory T Cells (Treg) with ^89^Zr-oxine for Medium-Term *In Vivo* Cell Tracking

**DOI:** 10.3390/molecules28031482

**Published:** 2023-02-03

**Authors:** Jacinta Jacob, Alessia Volpe, Qi Peng, Robert I. Lechler, Lesley A. Smyth, Giovanna Lombardi, Gilbert O. Fruhwirth

**Affiliations:** 1 MRC Centre for Transplantation, Peter Gorer Department of Immunobiology, School of Immunology and Microbial Science, King’s College London, Guy’s Hospital, Tower Wing, 5th Floor, Great Maze Pond, London SE1 9RT, UK; 2 Imaging Therapies and Cancer Group, Comprehensive Cancer Centre, School of Cancer and Pharmaceutical Sciences, King’s College London, Guy’s Campus, New Hunt’s House, 2nd Floor, Great Maze Pond, London SE1 1UL, UK; 3 School of Health, Sport and Bioscience, Stratford Campus, University of East London, London E15 4LZ, UK

**Keywords:** adoptive cell transfer, cell tracking, immunotherapy, PET/CT, transplantation, Zr-89-oxine

## Abstract

Regulatory T cells (Tregs) are a promising candidate cell therapy to treat autoimmune diseases and aid the longevity of transplanted solid organs. Despite increasing numbers of clinical trials using human Treg therapy, important questions pertaining to their *in vivo* fate, distribution, and function remain unanswered. Treg accumulation in relevant tissues was found to be crucial for Treg therapy efficacy, but existing blood-borne biomarkers are unlikely to accurately reflect the tissue state. Non-invasive Treg tracking by whole-body imaging is a promising alternative and can be achieved by direct radiolabelling of Tregs and following the radiolabelled cells with positron emission tomography (PET). Our goal was to evaluate the radiolabelling of polyclonal Tregs with ^89^Zr to permit their *in vivo* tracking by PET/CT for longer than one week with current preclinical PET instrumentation. We used [^89^Zr]Zr(oxinate)_4_ as the cell-labelling agent and achieved successful radiolabelling efficiency of human Tregs spanning 0.1–11.1 Bq ^89^Zr/Treg cell, which would be compatible with PET tracking beyond one week. We characterized the ^89^Zr-Tregs, assessing their phenotypes, and found that they were not tolerating these intracellular ^89^Zr amounts, as they failed to survive or expand in a ^89^Zr-dose-dependent manner. Even at 0.1 Bq ^89^Zr per Treg cell, while ^89^Zr-Tregs remained functional as determined by a five-day-long effector T cell suppression assay, they failed to expand beyond day 3 *in vitro*. Moreover, PET imaging revealed signs of ^89^Zr-Treg death after adoptive transfer *in vivo*. In summary, ^89^Zr labelling of Tregs at intracellular radioisotope amounts compatible with cell tracking over several weeks did not achieve the desired outcomes, as ^89^Zr-Tregs failed to expand and survive. Consequently, we conclude that indirect Treg labelling is likely to be the most effective alternative method to satisfy the requirements of this cell tracking scenario.

## 1. Introduction

Regulatory T cells (Tregs) represent a lymphocyte subset with immunosuppressive properties which is fundamental for the maintenance of tolerance to self-antigens *in vivo* [1,2]. Tregs readily migrate to sites of inflammation where they exert powerful anti-inflammatory properties and participate in tissue repair and regeneration. Tregs are characterized by a constitutively high expression of CD25 and the master transcription factor forkhead-box protein 3 (FOXP3). Tregs play pivotal roles in various autoimmune diseases [3,4,5], and they are crucial in solid organ transplantation, where they contribute to controlling responses to alloantigens. A correlation between the proportion of Tregs within a transplanted organ and graft survival has been observed [6,7,8]. This has led to the pursuit of protocols designed to tip the balance between Tregs and effector T cells (Teffs) in favor of Tregs to induce transplantation tolerance and cure autoimmune diseases such as type 1 diabetes. One strategy to elevate Treg numbers is the isolation and purification of Tregs from patients, expanding and potentially manipulating them *ex vivo*, and finally re-administering them to these patients. In humanized animal models transplanted with human skin and reconstituted with human peripheral blood mononuclear cells (PBMCs), adoptive transfer of human *in vitro* expanded polyclonal Tregs significantly prolonged the survival of skin allografts [9,10].

Polyclonal Treg-based cell therapy approaches yielded early promising results for the prevention of graft-versus-host disease, after allogeneic hematopoietic stem cell transplantation [11,12], and maintenance of C-peptide levels in type 1 diabetes [13,14]. We led two clinical phase I/II trials using adoptive transfer of polyclonal expanded Tregs to promote tolerance in kidney (ONE-Study: NCT02129881) and liver (ThRIL: NCT02166177) transplant recipients [15,16,17]. Resultant data suggested Treg therapy to be safe and well-tolerated and indicated signs of efficacy. Despite an increase in the numbers of clinical trials using human Treg therapy in transplantation and autoimmune diseases, important questions pertaining to their *in vivo* fate, distribution, and function remain unanswered. Recently, polyclonal Tregs labelled with [6,6-^2^H_2_] glucose were detected in the circulation of type 1 diabetic patients for up to one year [14]. Blood-borne parameters are unlikely to be relevant biomarkers reflecting the situation in relevant tissues; however, it remains a challenge to identify adoptively transferred Tregs within tissues non-invasively.

Cell tracking by whole-body imaging can address this challenge. Non-invasive radionuclide imaging by single photon emission computed tomography (SPECT) or positron emission tomography (PET) offers excellent sensitivity with absolute quantification and true 3D information while being translatable to clinical practice. Cell labelling is required to visualize and quantify cells by imaging. It can either be based on direct or indirect labelling, with each approach offering distinct advantages and disadvantages [18]. We previously showed that the direct radiolabelling of murine CD4^+^ T cells with ^99m^Tc-hexamethylpropyleneamine oxime did not affect cell viability, but the radiolabelled cells could only be detected for about 24 h due to the short half-life of ^99m^Tc (6.0 h) [19]. This allowed the assessment of Treg distribution within a day after administration. However, in transplant immunology, when aiming to track Tregs to transplants, even one week represents too short an observation time, and significantly longer cell tracking would be beneficial (several weeks).

Longer tracking times can be achieved either by indirect cell tracking based on genetically engineered cells or by using longer-half-life radioisotopes in a conventional direct cell-labelling approach [18,20]. A genetic-engineering-based methodology is, however, an unlikely contender when there is not already a need to genetically engineer the therapeutic cells for the purpose of conferring therapeutic efficacy. Consequently, for cell therapies that are not genetically engineered, there is a requirement to evaluate the feasibility of using longer-half-life radioisotopes for direct cell tracking. Longer-half-life radioisotopes are available, for example, ^111^In, ^89^Zr, and ^52^Mn with half-lives of 2.8, 3.3, and 5.6 days, respectively. However, they may also cause a higher received radioactive dose [21], thereby potentially risking study validity through unintended radio-damage to labelled therapeutic cells or radiation-associated side effects to healthy tissues. Using ^111^In-oxine-labelled Tregs, 72 h tracking was achieved clinically in humans [22]. Notably, cell labelling with the radioisotope ^89^Zr has been developed in recent years, because (i) ^89^Zr is a neutron-deficient radioisotope decaying *via* electron capture (77%) and positron emission (23%), thus being suitable for PET imaging, (ii) ^89^Zr presents with a half-life of 3.3 days, (iii) ^89^Zr, when complexed by ionophores such as 8-hydroxychinoline, is an efficient lipophilic cell-labelling agent [23] that readily labels many different cell types, and (iv) zirconium is a rare element in mammals, with no known biological function or relevant chemical toxicity [24]. Furthermore, various ^89^Zr-labelled T lymphocyte types were recently tracked for about one week in preclinical models [25,26,27], thereby providing a rationale for also evaluating its use in Tregs. So far, *in vivo* tracking of human Tregs beyond four weeks has not been achieved other than recently demonstrated by us through adopting a reporter gene engineering approach [28]. This work also pointed towards the relevant tracking times for Tregs in transplantation being longer than one week after administration. In principle, such tracking periods might also be within reach using direct cell labelling using ^89^Zr; however, ^89^Zr labelling under conditions permitting tracking times beyond one week in Tregs has not yet been evaluated.

Our goal here was to evaluate the radiolabelling of polyclonal Tregs with ^89^Zr at concentrations that could permit their *in vivo* tracking by PET/CT for longer than one week with current preclinical PET instrumentation.

## 2. Results

### 2.1. Radiolabelling of Tregs with ^89^Zr-oxine

**Figure 1:**
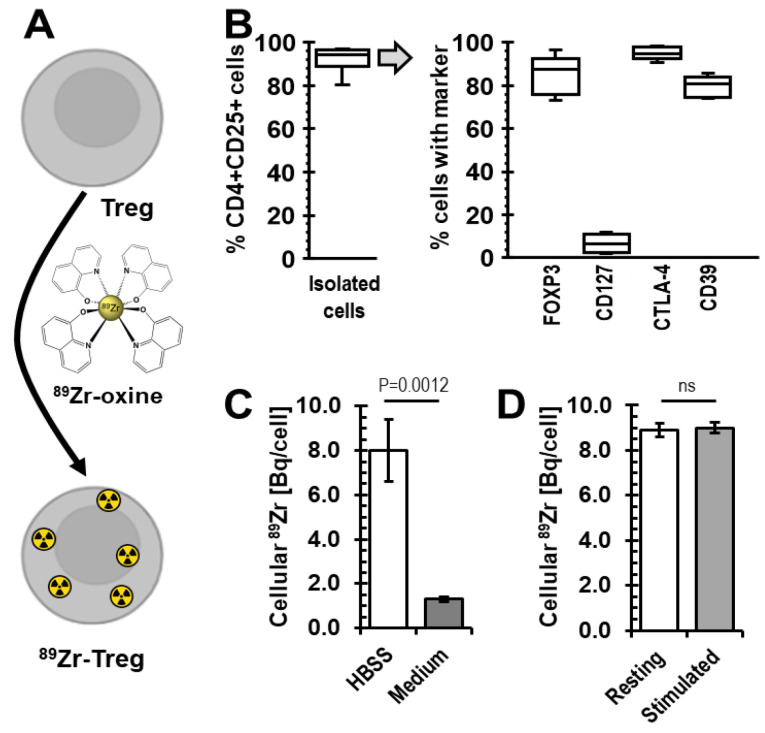
Human expanded Treg isolation and radiolabelling with ^89^Zr. (**A**) Scheme depicting the radiolabelling agent. (**B**) Isolated human expanded Tregs were analyzed by flow cytometry; the right panel shows expression of the CD4^+^CD25^high^ population from the left panel; *n* = 9 Treg isolations from different donors; box–whisker plot including the median line. For gating see Supplementary Figure S1. (**C**) Higher cellular ^89^Zr uptake was obtained when radiolabelling of Tregs with ^89^Zr-oxine was performed in saline compared to the optimal Treg growth medium (serum-free); *n* = 3 different Treg batches, error bars are SD. (**D**) We found no difference in radiolabelling efficiency when comparing resting and CD3/CD28-bead-stimulated Tregs; *n* = 3 different Treg batches, error bars are SD.

Building on our previous direct cell-labelling experience [19,23,26], we explored the feasibility of using [^89^Zr]Zr(oxinate)_4_ (^89^Zr-oxine) for Treg radiolabelling (Figure 1A). Human CD4^+^CD25^high^ CD127^low^FOXP3^+^ cells were enriched from donor blood using GMP-compatible protocols [15,29] and stimulated polyclonally *in vitro* with anti-CD3/CD28 beads in the presence of rapamycin and IL-2. After three weeks of culture, their phenotype was determined and their Treg nature confirmed (Figure 1B). Specifically, we found cells to be 92.1 ± 5.5% positive for both CD4 and CD25 markers and, when gated on this population, confirmed expression of Treg markers to be as expected (Figure 1B/right). Tregs were then radiolabelled with ^89^Zr-oxine as a cell-labelling agent to incorporate ^89^Zr into these cells.

It was previously reported that the radiolabelling efficiency of immune cells (dendritic cells, cytotoxic T cells, NK cells) with ^89^Zr-oxine depended on their culture medium [27]. Therefore, we investigated whether our regular Treg culture medium, X-Vivo15, provided an environment permitting good Treg labelling in comparison to saline, which previously served as a reference condition for cell labelling with oxinate-chelated radioisotopes. We found, however, that ^89^Zr-oxine labelling in Hank’s Balanced Salt Solution (HBSS) far outcompeted radiolabelling in X-Vivo15, with cellular Treg uptake being >4-times higher in HBSS (Figure 1C). Moreover, we evaluated whether resting or CD3/CD28-bead-stimulated Tregs were radiolabelled better in HBSS. Radiolabelling of resting Tregs did not differ significantly from radiolabelling of stimulated ones (Figure 1D).

**Figure 2:**
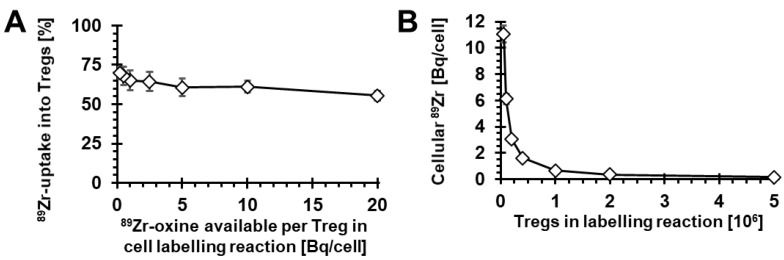
Variation of ^89^Zr-oxine-to-Treg cell numbers during radiolabelling. (**A**) The ^89^Zr-oxine amount per Treg cell in the labelling reaction had little effect on the fraction of ^89^Zr taken up into Tregs. (**B**) Intracellular ^89^Zr in Tregs after labelling increased with decreasing Teg numbers in the labelling reaction (concomitant with increasing ^89^Zr-oxine-to-Treg ratios). *n* = 3 and error bars represent SD. Note that total ^89^Zr-oxine in the reaction was constant as was Treg concentration in the reaction.

Next, we investigated the impact of increasing concentrations of ^89^Zr-oxine in relation to Treg numbers in the cell-labelling reaction. As Tregs are sensitive to their numbers in suspension during culture, we kept their cell concentration constant at 10^6^ cells/mL for all conditions. By keeping the absolute ^89^Zr-oxine amount (as determined by its radioactivity) constant for each labelling condition, we achieved variation of available ^89^Zr-oxine per Treg cell in the reaction over two orders of magnitude. Under these conditions, we found that radiolabelling was successful across all conditions, with approximately 55–70% of available ^89^Zr being taken up into Tregs (Figure 2A). As expected, the resultant cellular ^89^Zr uptake expressed as radioactivity per Treg increased with decreasing Tregs in the reaction. The resultant intracellular ^89^Zr per Treg ranged from 0.1 to 11.1 Bq/cell (Figure 2B).

### 2.2. Viability of ^89^Zr-labelled Tregs

**Figure 3:**
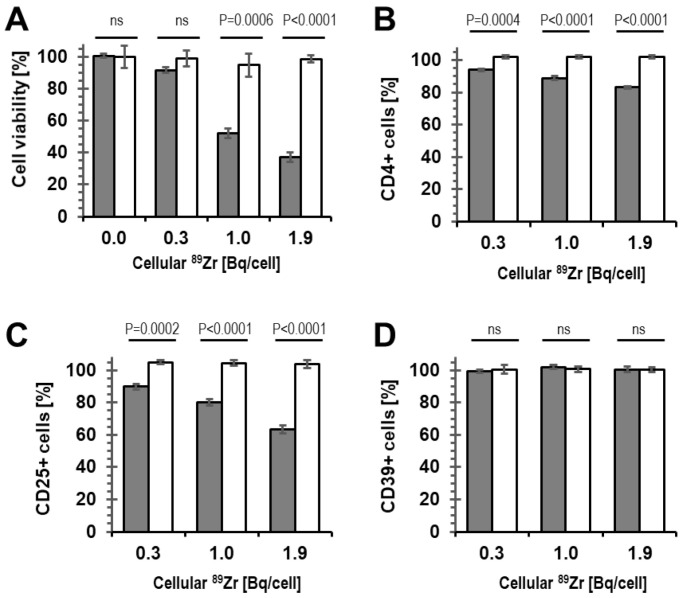
Viability and phenotype of ^89^Zr-labelled human Tregs compared to mock-labelled Tregs. (**A**) Treg viability assessed following 72 h of culture after radiolabelling. Tregs were analyzed by flow cytometry for the percentage of cells expressing (**B**) CD4, (**C**) CD25, and (**D**) CD39 markers. Data are normalized to untreated Tregs. (Gray) ^89^Zr-Tregs, (white) mock-labelled Tregs that do not contain ^89^Zr; *n* = 3 for all experiments with error bars representing SD.

Importantly, radiolabelling at high concentration can cause radio-damage to cells, which does not necessarily manifest in altered surface marker expression immediately after radiolabelling. Consequently, we assessed the viability of radiolabelled cells not straight after radiolabelling, but instead following three days of culture (in optimal growth medium). We found that radiolabelling resulting in cellular radioactivity >0.3 Bq ^89^Zr per cell negatively affected Treg viability, while at ≤0.3 Bq ^89^Zr per cell, we observed Treg viability of >90% (Figure 3A).

### 2.3. Phenotype and Suppressive Capacity of ^89^Zr-labelled Tregs

Furthermore, we were interested in the impact of cellular ^89^Zr on the phenotype of the radiolabelled Tregs. The percentage of CD4^+^ and CD25^+^ Tregs significantly decreased in the radiolabelled population compared to the control population, which had only received vehicle (DMSO) but no radioactivity during a mock cell-labelling reaction. Notably, this effect was observed even at intracellular ^89^Zr amounts that did not impact on Treg viability (*i.e.*, 0.3 Bq ^89^Zr/cell; compare Figure 3B,C with Figure 3A). No impact of radiolabelling with ^89^Zr on the percentage of CD4^+^CD25^+^ Tregs expressing CD39 was detected, not even at much higher cellular ^89^Zr amounts (up to 2 Bq/cell; Figure 3D). Together, this suggested that the presence of ^89^Zr within Tregs triggered downmodulation of CD4 and CD25 from the Treg plasma membrane. These results demonstrated that ^89^Zr-oxine labelling at amounts greater than 0.3 Bq/Treg cell resulted in phenotypic changes of the radiolabelled cells. Moreover, our results showed that assessment of cell viability alone (Figure 3A) was less sensitive to changes than phenotype analysis by CD4/CD25 surface marker quantification (Figure 3B,C).

**Figure 4:**
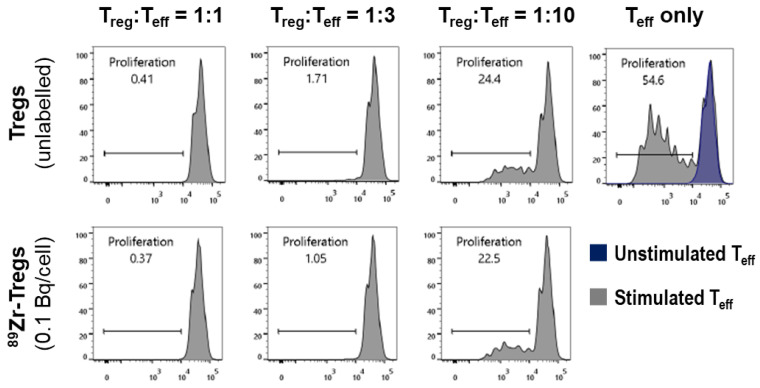
Suppressive capacity of radiolabelled ^89^Zr-Tregs. Dye-labelled effector T cells (Teffs) were allowed to proliferate in the absence (top right panel—gray: CD3/CD28-stimulated; purple: unstimulated) or presence of Tregs (all other panels). Teff proliferation was assessed from the reduction in intracellular fluorescent dye concentration, which reduced with every cell division and was quantified by flow cytometry. (**top**) Unlabelled Tregs and (**bottom**) ^89^Zr-Treg suppressed Teff proliferation in a dose-dependent manner. For this functional assay, ^89^Zr-Tregs were radiolabelled at 0.1 Bq/cell. Assay duration was 5 days. A typical example of *n* = 3 is shown.

Tregs are not only defined by their phenotype with respect to marker expression, but also by their function to suppress the proliferation of effector T cells (Teffs). We therefore determined whether ^89^Zr-oxine labelling had an impact on this Treg feature. We labelled HLA-A2-negative expanded Tregs with lower concentrations of ^89^Zr as in Figure 3 (due to the ^89^Zr impact observed there); we labelled them to amounts of 0.1 Bq ^89^Zr per Treg cell. We then labelled HLA-A2-positive freshly isolated Teffs with the CellTrace Violet dye and stimulated their proliferation with CD3/CD28-beads. Dye-labelled Teffs were combined with radiolabelled or unlabelled Tregs and co-cultured over five days, after which Teff proliferation and its suppression by Tregs were analyzed by flow cytometry [28,30]. Figure 4 shows corresponding histograms demonstrating both the CD3/CD28-bead-mediated Teff proliferation and its suppression by Tregs (in a Treg-dose-dependent manner). Notably, the Tregs used in this assay were added to the assay after radiolabelling; however, the assay does not represent a snapshot of Tregs’ suppressive capacity at assay start only, but instead a cumulative measurement over the assay duration of five days. These results were promising, as we did not find a negative impact of ^89^Zr labelling on Treg suppressive function at 0.1 Bq ^89^Zr/cell.

### 2.4. Survival of ^89^Zr-labelled Tregs in Immunodeficient Mice

**Figure 5:**
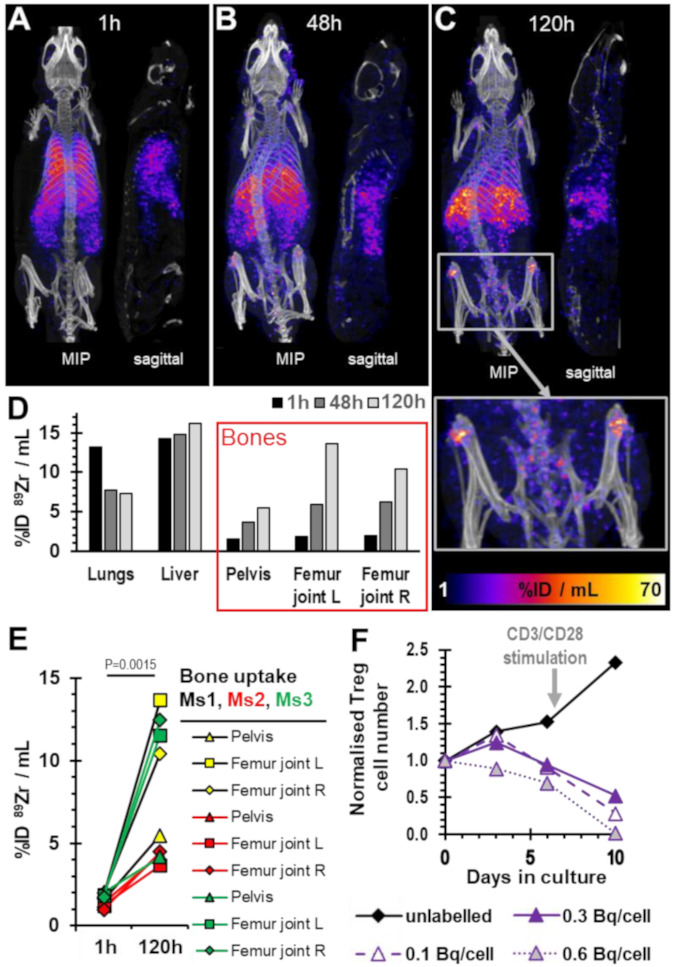
*In vivo* imaging of ^89^Zr-Tregs. (**A**–**C**) PET/CT overlay images of a representative BRG mouse that had received HLA-A2-negative ^89^Zr-Tregs (0.1 Bq/cell; 5 × 10^6^; 0.7 MBq total radioactivity administered) with HLA-A2-positive Teffs admixed (5 × 10^6^). Time points refer to the time passed after adoptive cell transfer. Maximum intensity projection images and sagittal sections of one of *n* = 3 mice are shown. The images are from the same mouse across (**A**–**C**). The inset in (**C**) shows 2-fold zoom of the pelvic area, clearly visualizing widespread and strong PET signals in various bones. (**D**) Quantitative ROI analysis of relevant tissues from the representative mouse shown in (**A**–**C**). (**E**) Comparison of three bone areas across three different mice imaged 1 h or 120 h after adoptive cell transfer. *P*-value calculated using two-tailed *t*-test (paired for each bone type in each mouse). *n* = 3 mice were used in this experiment and three different bone types analyzed per mouse. (**F**) The same ^89^Zr-Treg batch that was administered to mice was also analyzed *in vitro* for its expansion capability (solid line). Controls were unlabelled (black) and higher labelled (dashed, dotted lines) Tregs from the same donor. Immediately after day 6, all cultures were re-stimulated with CD3/CD28 beads (arrow).

Next, we were interested whether and for how long radiolabelled ^89^Zr-Tregs can be detected *in vivo* as well as how they would distribute in immunodeficient animals, as they are frequently the basis for establishing humanized mouse/xenograft models. Therefore, we labelled HLA-A2-negative expanded Tregs (95% of CD4^+^CD25^+^ cells positive for FOXP3^+^ in this batch) with ^89^Zr-oxine to levels of 0.1 Bq ^89^Zr/cell. A total of 5 million of these HLA-A2-negative ^89^Zr-Tregs were combined with 5 million human HLA-A2-positive CD25^−^ PBMCs and co-administered intravenously into BRG mice. The animals were imaged 1 h after adoptive cell transfer by PET/CT as well as 48 h and 120 h later. One hour post-administration, ^89^Zr-Tregs were found mainly in the lungs and liver, as was expected (Figure 5A). Two days later, ^89^Zr signals had decreased in the lungs, indicative of active ^89^Zr-Treg clearance from the lungs, which was also expected. ^89^Zr signals were at this point predominantly in the liver alongside minor signals in shoulder and knee joints (Figure 5B,D). Five days post-administration, although most radioactivity remained in the liver, large ^89^Zr signals were found in different bones, including the spine (Figure 5C,D; see also inset depicting the pelvic area). Quantitative image analysis showed ^89^Zr signals increased in various bones over time across different animals (Figure 5D,E). Importantly, Treg accumulation in bones is not expected. In contrast, ^89^Zr signals from bones are generally associated with free ^89^Zr that is rapidly mineralized [31].

Notably, we kept excess ^89^Zr-Tregs from this batch which were not administered to animals and assessed their growth *in vitro* alongside the animal experiment. This included various control Tregs: (i) mock-labelled Tregs that did not receive ^89^Zr as ‘expansion control’, (ii) ^89^Zr-Tregs labelled to 0.3 Bq/cell as controls, linking the experiments with previous ones in Figure 2 and Figure 3, and (iii) ^89^Zr-Tregs labelled to 0.6 Bq/cell as ‘damaged Treg control’. This experiment demonstrated that while ^89^Zr-Tregs radiolabelled to 0.1 and 0.3 Bq/cell initially expanded similarly to unlabelled Tregs (in line with data from Figure 3A), they failed to further expand (Figure 5F). This could not be rectified by re-stimulating the Tregs with CD3/CD28-beads (note higher expansion in mock-labelled Tregs after stimulation in Figure 5F), and all radiolabelled ^89^Zr-Tregs suffered from progressive cell death.

## 3. Discussion

Here, we aimed to radiolabel isolated and expanded human polyclonal Tregs with ^89^Zr for the purpose of non-invasive *in vivo* cell tracking beyond one week. It is noteworthy that several previous studies demonstrated successful radiolabelling of various immune cell types, but not Tregs, using ^89^Zr-oxine. For example, Sato *et al*. achieved ^89^Zr labelling of dendritic cells, cytotoxic T lymphocytes (CTLs), and NK cells at levels ranging from 0.009 to 0.045 Bq ^89^Zr per cell [27]. As for CTLs, they reported successful tumor therapy in the OT-1 tumor model with ^89^Zr-CTLs containing 0.032 Bq ^89^Zr per cell as well as *in vivo* tracking for seven days. Elsewhere, chimeric antigen receptor-engineered T cells were radiolabelled at 0.070 Bq ^89^Zr per cell, and *in vivo* tracking for around six days was reported [25]. We also ^89^Zr-labelled T cells before, more specifically, gamma-delta T cells at ~0.020 Bq ^89^Zr per cell, and tracked them non-invasively by PET over seven days in tumor-bearing mice [26].

Importantly, to achieve the longer *in vivo* cell tracking periods desired here, the intracellular ^89^Zr concentration per cell had to be increased. We built on two previous observations: (i) detection thresholds of current preclinical PET instrumentation permitted good determination of ^89^Zr-labelled cell distribution in mice one week after administration with 0.020–0.070 Bq ^89^Zr per cell, and (ii) ^89^Zr was generally well retained in T cells, and we assumed this was also the case for Tregs (for both observations, see [25,26,27]). As per the radioactive decay law, this meant 4.3-fold, 19.5-fold, or 86-fold higher amounts of intracellular ^89^Zr to obtain a similarly acceptable image quality after two, three, or four weeks of cell tracking, respectively (without considering label dilution and efflux). Building on these observations, we aimed for the intracellular ^89^Zr concentration in Tregs spanning ~0.2 to ~4.3 Bq ^89^Zr per cell. We achieved this through variation of ^89^Zr-oxine:Treg cell number ratios in the radiolabelling reactions, with obtained intracellular ^89^Zr concentrations ranging from 0.1 to 11.1 Bq ^89^Zr per Treg cell (Figure 2). Radiolabelling efficiencies were good in saline but poor in the widely used Treg growth medium X-Vivo15 (serum-free) (Figure 1C). While the detrimental effect of X-Vivo15 on radiolabelling was larger than reported with other immune cell media, our saline data are in line with others reporting the best ^89^Zr-oxine radiolabelling efficiencies under this condition [25,26,27,32]. Moreover, we found little impact of Treg activation status on radiolabelling efficiency (Figure 1D).

Treg viability assessed three days after radiolabelling was only unaffected by intracellular ^89^Zr at concentrations ≤0.3 Bq/cell (Figure 3A). It is noteworthy that even at 0.3 Bq/cell, the ^89^Zr-Treg phenotype, as defined by marker expression, was not stable, as the percentages of CD4^+^ and CD25^+^ cells in the expanded Treg population decreased following radiolabelling in a dose-dependent manner (Figure 3B,C). The study of the biological consequence of ^89^Zr radiation on Tregs was not the aim of this work, but phenotypic changes of Treg populations under distinct irradiation conditions have been described previously [33]. Functional assessment of Treg suppressive capacity was retained at radiolabelling with 0.1 Bq ^89^Zr per cell (Figure 4), which was therefore chosen as the only condition to evaluate ^89^Zr-Treg *in vivo* imaging in mice, despite it already being rather low compared to the needs for longer-term tracking (see above).

Importantly, the administered ^89^Zr-Tregs initially behaved *in vivo* as expected for intravenously administered cells, *i.e.*, they accumulated in the lungs at 1 h post administration and then largely cleared the lungs and redistributed elsewhere within the body within a day (Figure 5A–D). The liver accumulation after 24 h was expected, as it had been reported previously for both ^89^Zr-CTLs [27] and ^89^Zr-gamma-delta T cells [26]. In this context, it is further noteworthy that ^111^In-Tregs homed well to transplanted livers and spleens in a previous clinical trial [22].

The ^89^Zr signals recorded in bones, however, were unexpected to this extent. Moreover, they significantly increased over time across different bones (Figure 5). Importantly, bone uptake is indicative of free ^89^Zr [31], thereby pointing towards either ^89^Zr efflux from ^89^Zr:Tregs (and thereby the loss of the ability to track the Tregs) or the death of ^89^Zr-Tregs with concomitant release of free ^89^Zr. While small amounts of ^89^Zr efflux cannot be completely ruled out, it has previously been shown by Sato *et al*. that *in vitro* retention of ^89^Zr in resting CTLs was very good (~90% over several days). Notable reductions in intracellular ^89^Zr were predominantly linked to cell expansion/proliferation (*i.e.*, label dilution), and in their work there was also no obvious bone uptake detected over a week [27]. Concomitant with our *in vivo* work, we assayed expansion of Tregs and ^89^Zr-Tregs *in vitro* as well. This included the same batch that was administered *in vivo*, and additionally, differently labelled ^89^Zr-Treg batches made from the same human donor (Figure 5F). The experiments demonstrated the expected growth of ^89^Zr-Tregs, but only for the first three days (*cf.* Figure 5F: ^89^Zr-Tregs at 0.1 and 0.3 Bq/cell). Subsequently, the ^89^Zr-Tregs failed to further expand. The latter could also not be rectified by re-stimulation, and in fact, progressive cell death was observed over time (~40% cell loss compared to unlabelled Tregs already on day 6). Together, these data suggest that ^89^Zr-Tregs labelled at even 0.1 Bq/cell are unfit for tracking by PET, because their ability to expand/survive over several days and their ability to survive *in vivo* (as indicated by progressive ^89^Zr uptake into bones) appeared to be compromised. Tregs did not appear more tolerant to intracellular ^89^Zr than various effector T cells [25,26,27].

The consequence of this finding, however, is that Treg *in vivo* tracking over several weeks is not a viable strategy when employing the ^89^Zr approach and using existing PET instrumentation. The PET principle relies on positron–electron annihilation with consequential release and detection of 511 keV gamma photons. However, during its decay to ^89^Y, ^89^Zr additionally produces higher-energy gamma rays at 909 keV from ^89m^Y de-excitation [34]. This is different compared to the most frequently used PET isotope ^18^F, which does not present with these higher gamma rays but can only be exploited in cell tracking as part of radiotracers detecting reporter genes (due to its short 1.8 h half-life; see [35,36,37,38]). Here, we did not dissect which portion of the ^89^Zr irradiation was responsible for the observed damage to Tregs. A recently hyped long-half-life radioisotope, ^52^Mn, was also proposed for cell tracking. Unfortunately, it suffered from very large cell efflux across different cell types (irrespective of whether used as ^52^Mn-oxine or [^52^Mn]Mn-porphyrin [39]), and it presents with a complex decay also including high-energy gamma rays (936 keV and 1434 keV, among several others [40]); thus, ^52^Mn also appears unlikely to be a suitable alternative to ^89^Zr for Treg tracking. However, it cannot be ruled out that different PET radioisotopes with good cellular retention and half-lives in the range of days but with overall less harmful radiative decay than ^89^Zr may permit somewhat higher intracellular radioisotope concentrations, perhaps allowing for some elongation of the observation time with current PET instrumentation. As increasing the intracellular amount of ^89^Zr was not tolerable to Tregs, the only remaining option to extend the observation time when using ^89^Zr could come from technological advancements in PET instrumentation, whereby at least an order of magnitude gain in sensitivity would be required to provide a meaningful extension of tracking time to enable Treg tracking over several weeks. This situation is likely similar when attempting to track other ^89^Zr-labelled T cell types over weeks, as well. Once such technical advancements have come to fruition, it might be worthwhile to revisit the question of how long T lymphocytes may be trackable *in vivo* at safe intracellular ^89^Zr concentrations.

To conclude, ^89^Zr labelling of Tregs at intracellular radioisotope amounts compatible with cell tracking over several weeks did not achieve the desired outcomes. Currently, the best alternative for longer-term tracking of T lymphocytes is indirect cell tracking relying on non-immunogenic host reporter genes that are engineered into the cells of interest. Several options exist that exploit short-half-life radiotracers and employ repeat imaging for cell tracking [18]. The latter concept has been demonstrated for Tregs [28] and currently represents the best way to track Tregs for observation intervals extending beyond one week.

## 4. Materials and Methods

**Reagents.** Standard reagents were from Corning, Merck, Sarstedt, Thermo-Fisher, or VWR. ^89^Zr was purchased from Perkin Elmer in 0.1 M oxalic acid and converted to the cell-labelling agent [^89^Zr]Zr(oxinate)_4_ (^89^Zr-oxine) as previously described [23].

**Purification and culture of human PBMCs, Tregs and Teffs.** Cells were isolated from peripheral blood donated by anonymous healthy volunteers *via* the UK National Health Services Blood Transfusion Service (NHSBT) with informed consent (available upon request from NHSBT) according to our GMP protocols [15,16,17]. Peripheral blood mononuclear cells (PBMCs) were separated by Lymphoprep density gradient centrifugation (PAA). CD4^+^ T cells were enriched using RosetteSep (StemCell Technologies) followed by selection and separation of CD4^+^CD25^+^ Tregs and CD4^+^CD25^−^ Teffs using CD25 microbeads (Miltenyi, Woking). PBMCs were depleted of CD25^+^ cells also by using CD25 microbeads (Miltenyi, Woking) and are referred to as CD25^-^ PBMCs. Aliquots of CD25^-^ PBMCs, Tregs, and Teffs were cryopreserved in 5% (*v*/*v*) human serum (BioSera, Heathfield) containing 10% (*v*/*v*) DMSO. Tregs were activated with anti-CD3/CD28 beads (1:1 bead:cell ratio; Miltenyi, Woking) and cultured in X-Vivo15 medium (Lonza) supplemented with both 5% (*v*/*v*) human AB-serum (BioSera, Heathfield; Treg medium) and 100 nM rapamycin (LC-Laboratories, Woburn/MA, USA). A total of 1000 U/mL recombinant human IL-2 (Proleukin from Clinigen, London) was added 72 h after cell isolation. After 10 d, Tregs were activated with anti-CD3/CD28 beads (1:1 bead:cell ratio) for further expansion for 10 d. X-Vivo15 media supplemented with both human IL-2 and rapamycin (see above) was used to feed the cells every other day when their concentration was adjusted to 10^6^/mL. Tregs were cultured at 37 °C in the presence of 5% (*v*/*v*) CO_2_ in a humidified incubator.

**Treg radiolabelling.** Isolated and expanded Tregs were re-suspended at 1 × 10^6^/mL in HBSS or X-Vivo15 as indicated and radiolabelled at room temperature (RT) through addition of 1 MBq ^89^Zr-oxine in aqueous DMSO (≤ 0.5% (*v*/*v*)). Notably, as the Treg concentration remained constant to ensure Tregs did not become too dilute (thereby potentially negatively affecting them), the volume of the Treg suspension to which we admixed ^89^Zr-oxine was varied (to achieve different radiolabelling conditions). After 20 min incubation, cells were pelleted, supernatant was collected, and cells were washed twice with 1 mL sterile X-Vivo15 before cells were resuspended in fully supplemented X-Vivo15 growth medium for γ-counting and further culture/experiments. ^89^Zr uptake into cells was determined by quantifying radioactivity in cells and pooled supernatant/wash solutions using a γ-counter (1282-Compugamma, LKB-Wallac, Mt Waverly, Australia) and calculated using Equation (1) (wherein Cpm represents decay-corrected radioactivity counts per minute).

(1) $$89^{\;}Zr\;uptake\;\left[\%\right]=\frac{Cpm\;\left[Cells\right]}{Cpm\;\left[Cells\right]+Cpm\;\left[Supernatant\;and\;Washes\right]}\cdot 100$$

Intracellular ^89^Zr was derived thereof by converting to Bq and dividing by the Treg number present, and data are expressed as Bq/cell.

**Determination of cell viability.** Viability was assessed using the LIVE/DEAD Fixable Near-IR Dead Cell Stain Kit (ThermoFisher, Loughborough, UK) according to manufacturer’s recommendations. Briefly, cells were resuspended in 1:1000 diluted LIVE/DEAD staining solutions and incubated for 30 min at 4 °C, followed by two PBS-washes, resuspension in 150 μL FACS buffer, and flow cytometric analysis.

**Flow cytometric marker analysis.** Cells were stained in phosphate-buffered saline (PBS) supplemented with 1% (*v*/*v*) FCS/5 mM EDTA using fluorescently conjugated primary antibodies at concentrations ranging from 2–5 µg/mL. Antibodies used were specific for CD4 (clone OKT4; conjugated to BV496; BD Bioscience, Worthing), FOXP3 (PCH101; eFLuor660; eBioscience now ThermoFisher, Loughborough), HLA-A2 (BB7.2; PE-Cy7; eBioscience), CD39 (eBioA1; PE-Cy7; eBioscience), CD25 (2A3; BV605; BD Bioscience), CTLA-4 (BNI3; BV421; BD Bioscience), and CD127 (A019D5; BV786; BioLegend). Only live cells were analyzed, with dead cells being excluded based on counterstaining with a near-infrared dye (LIVE/DEAD Fixable Near-IR Dead Cell Stain kit; ThermoFisher, Loughborough). Intracellular staining was performed using the Fix/Perm kit (eBioscience). Data were acquired using an LSRFortessa II (BDBioscience) and analyzed using FlowJo v.7 (FlowJo, Ashland/OR, USA).

**Suppression assay.** A total of 10^7^ cells/mL HLA-A2-positive CD4^+^CD25^−^ effector T cells were resuspended in staining solution containing 5 µM CellTrace Violet (CTV; ThermoFisher) and incubated at 37 °C for 15 min, twice washed in complete growth medium, and resuspended in growth medium (at 10^6^ Teffs/mL). HLA-A2-negative Tregs were harvested from culture, and activation beads were removed *via* magnetic separation, with cells washed in complete growth medium and resuspended in growth medium (at 10^6^ Tregs/mL). A total of 10^5^ CTV-labelled Teffs were plated in 96-well ‘U-bottom’ plates, and Tregs were added for co-culture (37 °C, 5% (*v*/*v*) CO_2_) at indicated ratios. Dynabeads Human T-Activator CD3/CD28 beads (ThermoFisher) were added at a bead:cell ratio of 1:40. Controls included non-activated Teffs and activated Teffs in the absence of Tregs. Teff proliferation was assessed after five days. Therefore, cells were harvested and stained with anti-human HLA-A2 (2 μg/mL) for 15 min at 4 °C to distinguish between Teffs and Tregs. Using flow cytometry (BD Fortessa cell analyzer), gates were placed on Teffs and their CTV profiles determined to quantify their proliferation. Results are shown as %suppression (inverse of %Teff proliferation) relative to Teffs cultured alone. It is noteworthy that this assay, which spans five days of co-culture, determines cumulative suppressive effects exerted by the Tregs over the whole assay duration.

**Animals.** BALB/c recombination activating gene 2 (Rag2)-/-γc-/- (BRG; gift from A. Hayday, King’s College London) mice were bred at licensed in-house animal facilities. All mice used were male and between 6 and 8 weeks old at the beginning of the experiment. Three mice were used for experiments in Figure 5. Mice were maintained within the King’s College London Biological Services Unit under specific pathogen-free conditions in a dedicated and licensed air-conditioned animal room (at 23 ± 2 °C and 40–60% relative humidity) under light/dark cycles lasting 12 h every day. They were kept in individually ventilated standard plastic cages (IVC; 501 cm^2^ floor space; from Tecniplast UK, London) including environmental enrichment and bedding material in the form of sterilized wood chips, paper stripes, and one cardboard roll per cage. Maximum cage occupancy was five animals, and animals were moved to fresh cages with fresh environmental enrichment and bedding material twice per week. Sterilized tap water and food were available ad libitum; food was PicoLab Rodent Diet 20 (LabDiet) in the form of 2.5 × 1.6 × 1.0 cm oval pellets that were supplied at the top of the cages. For imaging, animals were anesthetized using isoflurane (1.5% (*v*/*v*) in pure O_2_). After imaging, mice were either left to recover from anesthesia (by withdrawal of anesthetic) in a pre-warmed chamber or sacrificed under anesthesia by cervical dislocation. No adverse events were associated with the procedures performed in this study, and animals put on weight in line with strain expectations (data from Charles River UK) throughout. Sentinel animals were kept on the same IVC racks as experimental animals and confirmed to be healthy after completion of the studies.

***In vivo* imaging of ^89^Zr-Tregs.** Male BRG mice (8 weeks old) were anesthetized with 2% (*v*/*v*) isoflurane/O_2_. Animals then received 5 million ^89^Zr-Tregs (0.1 Bq ^89^Zr per cell) co-administered with 5 million HLA-A2-negative CD25^-^ PBMCs into their tail veins, reflecting conditions that were previously established and optimized [28,30]. The total amount of radioactivity administered was 0.7 MBq ^89^Zr. Animals were kept under anesthesia after adoptive cell transfer and placed in the sphinx position onto animal supports for PET/CT imaging (NanoScan PET/CT; Mediso, Farnborough). A total of 45 min after ^89^Zr-Treg administration, CT images were acquired (55 kVp tube voltage, 1200 ms exposure time, 360 projections), and 60 min after adoptive cell transfer, PET images were acquired (30 min scans). PET/CT data were reconstructed using a Monte-Carlo-based full-3D iterative algorithm (Tera-Tomo; Mediso, Farnborough) with corrections for attenuation, detector dead time, and radioisotope decay in place as needed. All images were analyzed using VivoQuant software (inviCRO Ltd., Doylestown/PA, USA) enabling the delineation of regions of interest (ROIs) for quantification of activity. CT images were used to draw ROIs for PET signal quantification. PET (hue) and CT (grayscale) images were overlayed for better appreciation of anatomical context of PET signals. The total activity in the whole animal (excluding the tail) at the time of tracer administration was defined as the injected dose (ID). Image-based ROI analysis of relevant organs was performed, and radioactivity data were expressed as %ID/mL. 

**Statistical analysis** was performed using Prism Software v9 (GraphPad Software Inc.). Differences between two groups were evaluated by Student’s *t*-test (two-tailed). Comparison of late with early time points in the animal study was performed, treating data as paired when from the same animal/organ. Details to the meaning of error bars and repeats are described in the figure legends.

**Ethical statement.** The study involved human samples as well as animals. Human blood was obtained with ethics approval (institutional review board reference 09/H0707/86). All procedures related to animal work were performed in accordance with all legal, ethical, and institutional requirements in the UK (under authority of the HO project license PPL70/7302); animal use was minimized in line with the latter requirements.

## Data Availability

The data presented here are available on request from the corresponding author. The animal imaging data are not publicly available due to a lack of repository and the size of the raw original data sets.

## Supplementary Materials

According to UK research councils’ Common Principles on Data Policy, all data supporting this study will be openly available at https://doi.org/10.5281/zenodo.7415485 (accessed on 14 December 2022).

## Sample Availability

Tregs were isolated from anonymized human donors. Animals were in-house bred. All compounds and reagents used were commercially available.
